# Genome-wide analysis and identification of the low potassium stress responsive gene *SiMYB3* in foxtail millet (*Setariaitalica* L.)

**DOI:** 10.1186/s12864-019-5519-2

**Published:** 2019-02-15

**Authors:** Xinyou Cao, Liqin Hu, Xueyan Chen, Rongzhi Zhang, Dungong Cheng, Haosheng Li, Zhaoshi Xu, Liancheng Li, Yongbin Zhou, Aifeng Liu, Jianming Song, Cheng Liu, Jianjun Liu, Zhendong Zhao, Ming Chen, Youzhi Ma

**Affiliations:** 1 Crop Research Institute, Shandong Academy of Agricultural Sciences/National Engineering Laboratory for Wheat and Maize/Key Laboratory of Wheat Biology and Genetic Improvement in North Yellow and Huai River Valley, Ministry of Agriculture, Jinan, 250100 People’s Republic of China; 2 National Key Facility for Crop Genetic Resources and Genetic Improvement, Key Laboratory of Crop Genetics and Breeding, Ministry of Agriculture/Institute of Crop Sciences, Chinese Academy of Agricultural Sciences, Beijing, 100081 People’s Republic of China; 3 Biotechnology Research Center, Shandong Academy of Agricultural Sciences/Shandong Provincial Key Laboratory of Crop Genetic Improvement, Ecology and Physiology, Jinan, 250100 People’s Republic of China

**Keywords:** Foxtail millet, Potassium, Transcriptome, RNA sequencing

## Abstract

**Background:**

Potassium (K) is essential to plant growth and development. Foxtail millet (*Setaria italic* L.) is an important fodder grain crop in arid and semi-arid regions of Asia and Africa because of its strong tolerance to drought and barren stresses. The molecular mechanisms of physiological and biochemical responses and regulations to various abiotic stresses such as low potassium conditions in foxtail millet are not fully understood, which hinders the research and exploitation of this valuable resource.

**Results:**

In this research, we demonstrated that the millet variety Longgu 25 was the most insensitive variety to low potassium stress among other five varieties. The transcriptome analysis of Longgu 25 variety revealed a total of 26,192 and 26,849 genes from the K^+^-deficient and normal transcriptomic libraries by RNA-seq, respectively. A total of 1982 differentially expressed genes (DEGs) were identified including 866 up-regulated genes and 1116 down-regulated genes. We conducted a comparative analysis of these DEGs under low-K^+^ stress conditions and discovered 248 common DEGs for potassium deprivation among foxtail millet, rice and Arabidopsis. Further Gene Ontology (GO) enrichment analysis identified a series of candidate genes that may involve in K^+^-deficient response and in intersection of molecular functions among foxtail millet, rice and Arabidopsis. The expression profiles of randomly selected 18 candidate genes were confirmed as true DEGs with RT-qPCR. Furthermore, one of the 18 DEGs, *SiMYB3*, is specifically expressed only in the millet under low-K^+^ stress conditions. Overexpression of *SiMYB3* promoted the main root elongation and improved K^+^ deficiency tolerance in transgenic Arabidopsis plants. The fresh weight of the transgenic plants was higher, the primary root length was longer and the root surface-area was larger than those of control plants after K^+^ deficiency treatments.

**Conclusions:**

This study provides a global view of transcriptomic resources relevant to the K^+^-deficient tolerance in foxtail millet, and shows that *SiMYB3* is a valuable genetic resource for the improvement of K^+^ deficiency tolerance in foxtail millet.

**Electronic supplementary material:**

The online version of this article (10.1186/s12864-019-5519-2) contains supplementary material, which is available to authorized users.

## Background

Potassium (K) is one of the essential macronutrients required for plant growth and development and involves in many important physiological processes in plant cells, including osmoregulation, turgor pressure control, electrical neutralization, and enzyme activation [1]. Previous studies have reported that K^+^ deficiency is a key abiotic stress factor in plants [2–4]. In soil, the K^+^ concentration is generally within the range of 1 to 200 ppm (approx. 0.025 ~ 5 mM) [5]. However, the K^+^ concentration in the rhizosphere is usually less than 0.3 mM, and K^+^ is deficient in large areas of cultivated land [6, 7]. Therefore, most plants are subjected to low K^+^ stress at some points during their development. Plants possess multiple K^+^ uptake systems (transporters and channels) with different K^+^ affinities and transportation activities to absorb adequate amounts of K^+^ from soil. In general, K^+^ channels mediate low-affinity K^+^ uptakes, and K^+^ transporters conduct high-affinity K^+^ uptakes. Transcriptional regulation is an important mechanism of plants in response to low-K^+^ stress [8–10]. For example, the expressions of several members of the KUP/HAK/KT family *AtHAK5* [11], *AtKUP3* [12], *OsHAK1* [13], *HvHAK1* [14, 15], as well as K^+^ transporter genes *AtCHX17* [16] and *TaAKT1* [17] were induced by K^+^ starvation. These K^+^ transporters may involve in K^+^ acquisition and homeostasis in plant cells under low-K^+^ conditions. Furthermore, transcriptomic analysis of Arabidopsis plants under low-K^+^ stress identified many candidate genes related to low-K^+^ perception and regulatory pathways associated with the response to K^+^ deficiency [18]. In rice, *OsAKT1* is the main inward rectifying K^+^ channel in roots [19] and contributes considerably to K^+^ uptake at a wide range of external K^+^ concentrations, overexpression of *OsAKT1* enhanced K^+^ uptake, which is beneficial in both K^+^-deficient and water-stress conditions [20]. Although considerable work has been done to understand the mechanisms of the plant response to K^+^ deficiency, an extensive work is still needed in crop plants to understand the detail mechanisms of K^+^ nutrition and signaling.

Foxtail millet *Setaria italic* (Poales, Poaceae) is an important C4 panicoid crop in arid and semi-arid regions of Asia and Africa due to its strong tolerance to drought and barren stresses. In addition, it possesses several salient attributes such as small genome (~ 515 Mb; 2n = 2×=18) with a relatively lower repetitive DNA, an inbreeding nature and a short life cycle. Its genome has been sequenced by two independent groups namely the Beijing Genomics Institute (BGI) and China-US Department of Energy-Joint Genome Initiative (JGI) [21, 22]. So, foxtail millet has become an ideal material of monocotyledon to study the mechanisms of plant stress tolerance [23]. The availability of genome sequences in public databases has encouraged the foxtail millet research community to perform high throughput investigations in the aspects of functional genomics [24–26]. There are about 27,059 catalogued accessions germplasm resources [27]. However, they are not widely used in the functional analysis of important genes, particularly, in the research related to the functional genomics of K^+^ sensing, uptake, distribution and homeostasis in foxtail millet. As monocotyledon, it is meaningful to compare transcriptomic changes among foxtail millet, rice and Arabidopsis under low-K^+^ stress to gain insights into which mechanisms are shared and which are specific to each plant species.

RNA-Seq technology has already been broadly utilized in the study of differential gene expression during plant responses to various biological and nonbiological stresses [28]. In the present study, we report such comparative RNA-seq study for the first time to further understand the complexity of foxtail millet under K^+^ deficiency at the whole transcriptome level. We found that there is a significant difference in K^+^ deficiency tolerance between 6 foxtail millet varieties, which have different characteristics of tolerance levels to drought and barren stresses and a wide adaptability in China, and discovered a K^+^ efficient variety Longgu 25. Then, we used RNA-Seq technology to monitor the transcriptomic profiles of the foxtail millet variety in responses to K^+^ deficiency. We analyzed the functional categorization of differentially expressed genes and compared the low-K^+^ response profiles between normal and K^+^ deficiency treatments. Using the published data, we compared the differences in the gene expression profiles of rice, Arabidopsis and foxtail millet under potassium deprivation, and found 248 common genes and 1734 foxtail millet specific genes. Overexpression of one of these millet specific genes *SiMYB3* in Arabidopsis enhanced K^+^ deficiency tolerance.

## Results

### Phenotypic analysis of foxtail millet seedlings under K^+^ deficiency

**Fig. 1:**
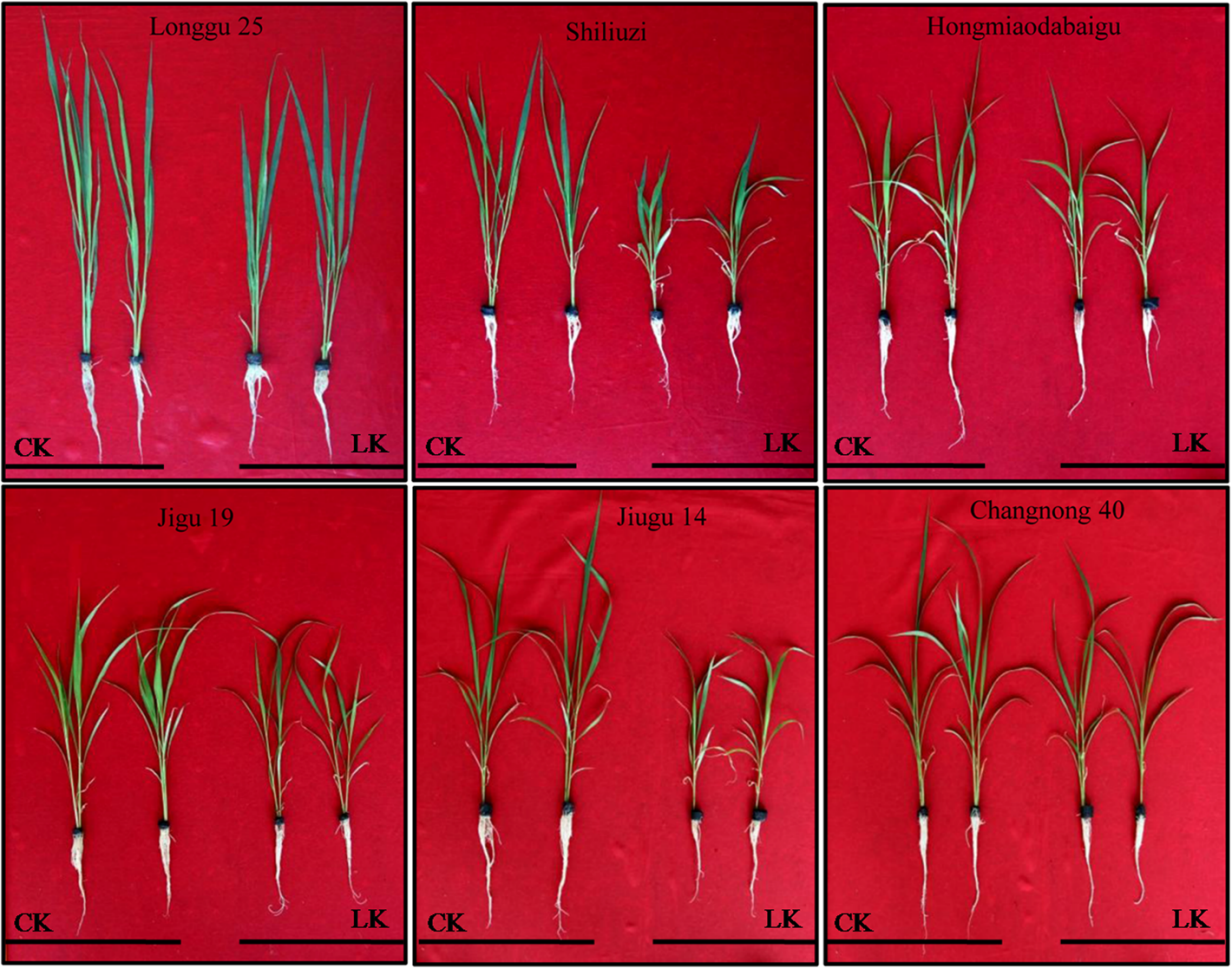
Differential responses of six foxtail millet varieties to K^+^ deficiency during seedling growth stage. The seedlings were transferred to modified Hoagland nutrient solution with 10 μM K as the K^+^-deficient treatment (LK) supplemented with NH_4_Cl as the nitrogen source. The plants grown in normal media (CK) are shown on right and the plants experienced K^+^ deficiency (LK) are shown on left

The 4-leaf stage seedlings had reduced shoot and root growth and leaf blade height for most varieties of six foxtail millet varieties after they were grown in potassium deficient media (LK) for 10 days. The lowest shoot-root ratio is 4.26 (Table 1). However, there was no phenotypic difference for Longgu 25 variety (Fig. 1). The shoot-root ratio of Longgu 25 was 6.01, the highest among all varieties (Table 1). There was a significant difference in total dry weight and the ground biomass between different varieties. The relative index (X_LK_/X_CK_) of total dry weight (0.87) and the ground biomass (0.85) of Longgu 25 was the highest in all varieties (Table 1). There was no significant difference in the shoot K^+^ content between different varieties after the LK stress treatment (Table 1). So, Longgu 25 was selected for further study as the tolerant variety for K^+^ deficiency.

**Table 1:**
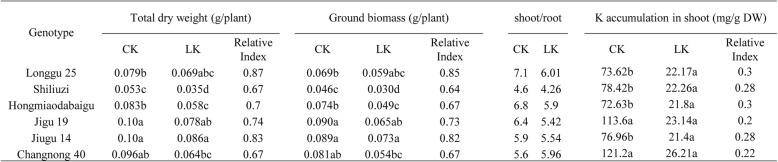
Total dry weight, the ground biomass and K^+^ content in shoot of different varieties in the K^+^ deficient (LK) and normal condition

### RNA-Seq results and mapping of the sequence reads

To obtain a global view of transcriptome profiles relevant to the K^+^-deficient treatment in Chinese foxtail millet variety Longgu 25, the high throughput RNA-Seq analyses on poly(A^+^)-enriched RNAs from the untreated (CK) and LK libraries were performed using the Solexa/Illumina platform. After filtering out the low-complexity reads, the low-quality reads and the repetitive reads, 26,666,736 usable reads for the CK plants and 26,665,015 usable reads for the LK plants were obtained (Table 2). Of the total reads, 71.47% of the LK reads matched to unique (54.92%) or multiple (16.55%) genome locations, however, 77.34% of the CK reads matched to unique (44.27%) or multiple (33.07%) genome locations (Table 2). As listed in Table 2, approximately 40.17% of the LK reads and 31.27% of the CK reads were mapped to unique genes, and 16.53% of the LK reads and 12.49% of the CK reads were mapped to multiple reference genes (Additional file 1: Table S2).

**Table 2:**
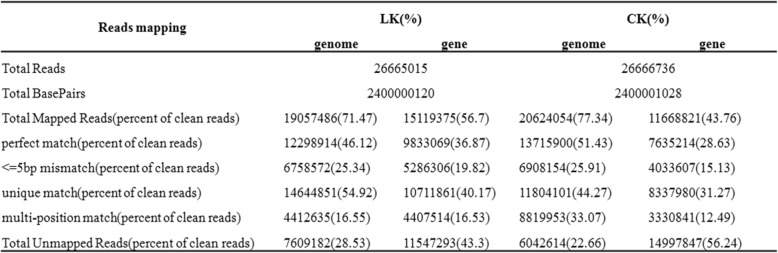
Number of assembly reads sequenced and mapped to the genome and genes

### Transcriptome analysis of the millet variety Longgu 25 in response to potassium deficiency stress

Correlation analysis of gene expression levels among samples is the key criterion to test whether the results are reliable and the collection of samples is reasonable. Based on the FPKM results, the correlation efficiencies among three replicates were calculated, where the square of the correlation efficiencies was greater than 0.93. Results showed that the experiment has good repeatability. To check if there is any difference in the gene expression, which may affect the phenotypic differences observed under K^+^ deficient conditions, we compared the RPKM-derived read counts and found 1982 genes expressed differentially between CK and LK samples, among them 866 up-regulated and 1116 down-regulated under the LK condition (Additional file 1: Table S3).

**Fig. 2:**
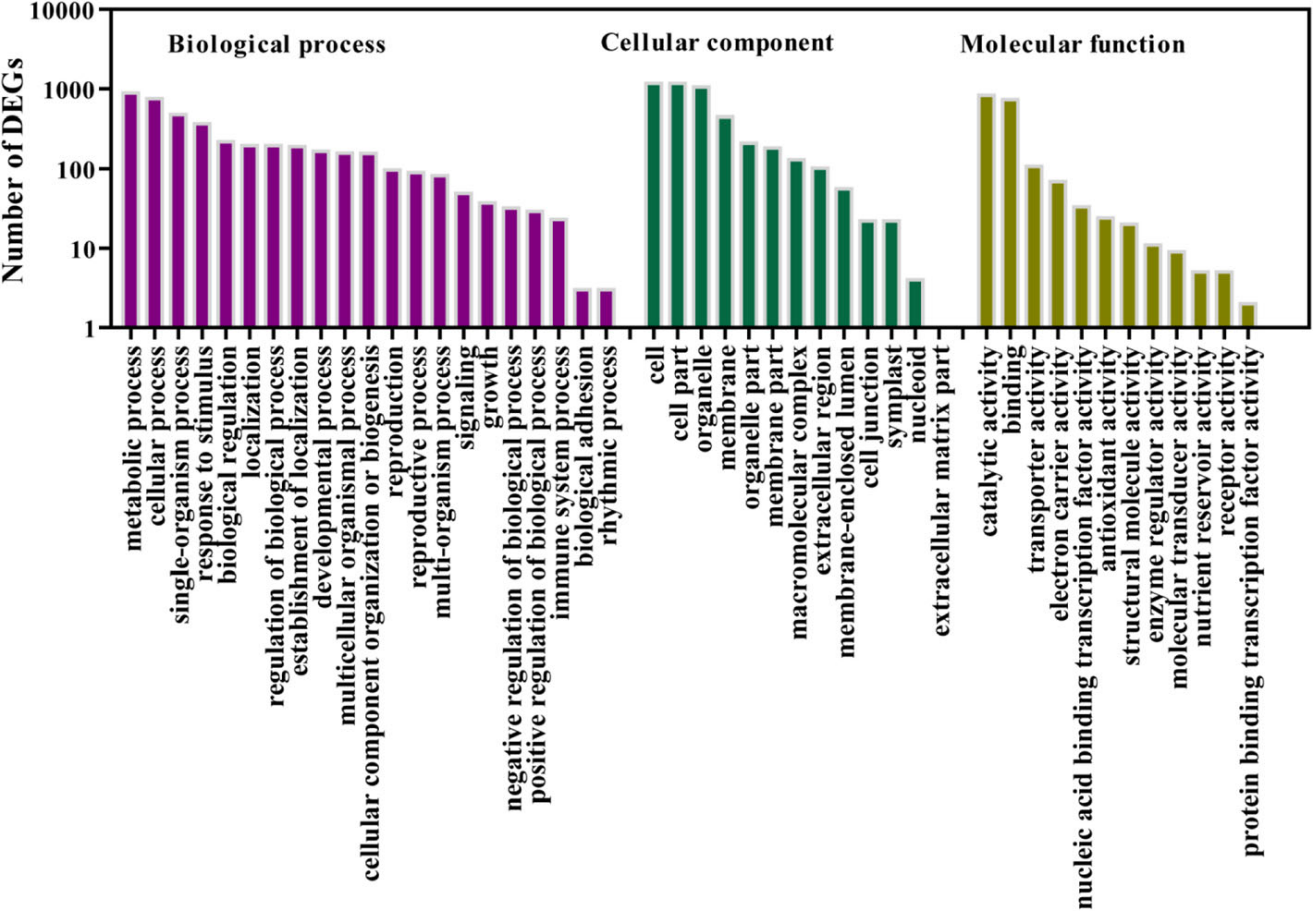
Gene Ontology (GO) functional annotation of DEGs. All transcripts were assigned to at least one GO term and grouped into three main GO categories and 46 subcategories, including 21 categories in biological process, 13 in cellular component, and 12 in molecular function. The Y-axis represents the number of genes in a sub-category

To evaluate potential functions of these differentially expressed genes (DEGs) in response to K^+^ deficiency, Gene Ontology (GO) analysis was performed by mapping each DEG into the records of the GO database (http://www.geneontology.org/). Under the biological process category, 21 GO categories were significantly enriched (corrected *p*-value≤0.05) (Fig. 2; Additional file 1: Table S4). Many genes that respond to various processes and stimuli were prominently represented, suggesting that these processes and stimuli may relate to the response of K^+^ deficiency. For the cellular component category (13 GO categories) (Fig. 2; Additional file 1: Table S4), most of the DEGs were associated with cell, cell part, organelle and membrane. Under the category of molecular function (12 GO categories) (Fig. 2; Additional file 1: Table S4), the main functional groups of the DEGs were genes with catalytic activity and activities of binding, transporter and nucleic acid binding of transcription factor (TF).

**Fig. 3:**
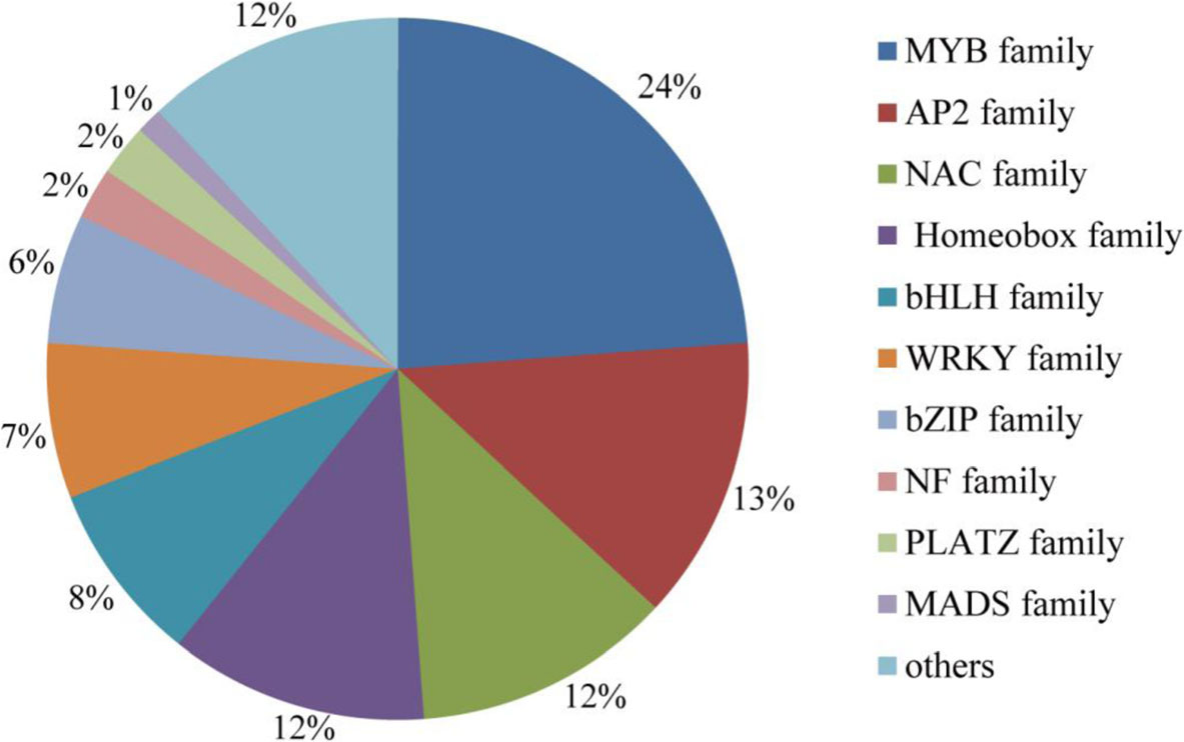
Distribution of transcription factor families differentially expressed in foxtail millet seedlings under the K^+^ deficient condition

Interestedly, we detected the differential expression of 84 genes encoding for TFs under K^+^ deficiency conditions in foxtail millet, including 41 up-regulated and 43 down-regulated TF genes (Fig. 3 and Additional file 1: Table S5). TFs are the regulatory proteins, which play a key role in stress responses. Among the various families, the largest number of differentially expressed TFs was in the v-myb avian myeloblastosis viral oncogene homolog (MYB) TF family (24%). The members of other TF families, including APETALA2 (AP2) (13%), NAM/ATAF1/2/CUC1/2 (NAC) (12%), Homeobox (12%), basic Helix-Loop-Helix (bHLH) (8%), WRKY (7%) and basic region leucine zipper (bZIP) (6%), were also highly represented in the DEGs (Additional file 1: Table S5).

### KEGG analysis

**Fig. 4:**
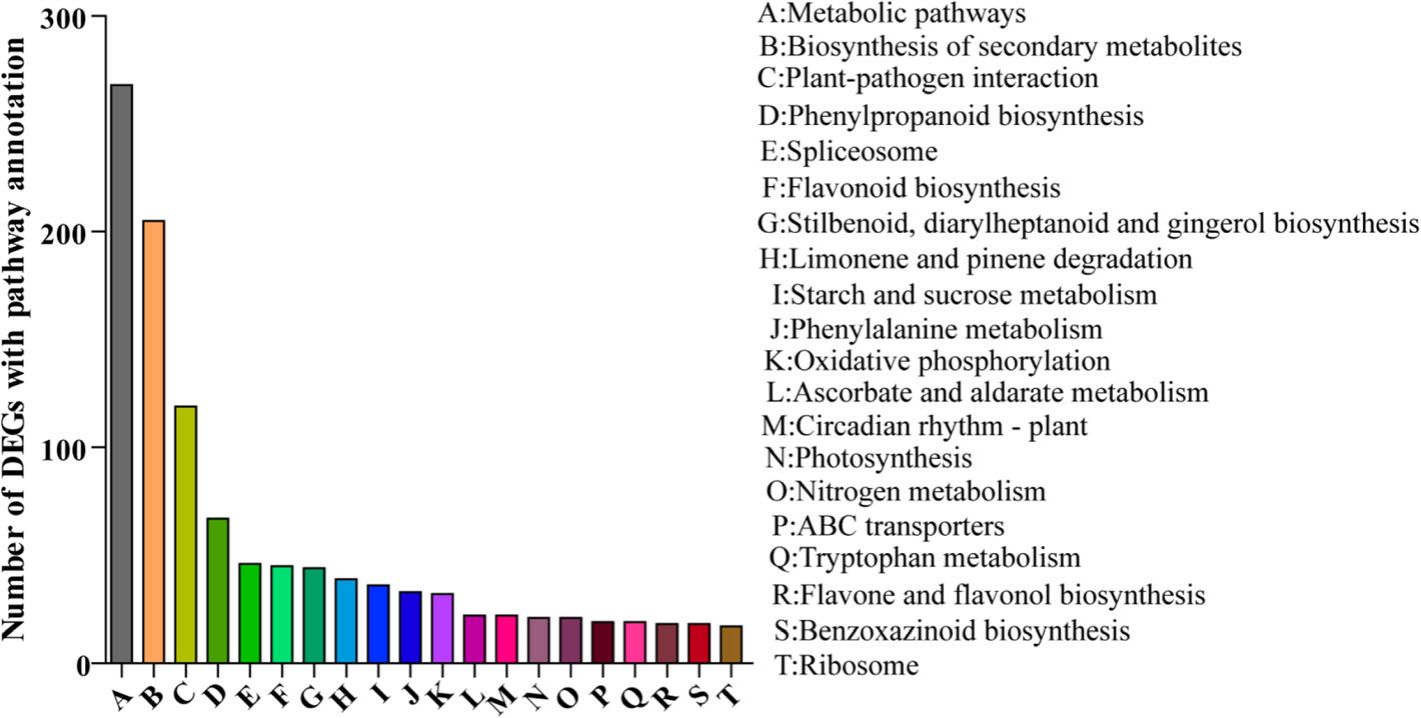
DEG enrichment analysis of 20 (A-T) main pathways under low potassium stress. The x-axes represent the category of each pathway. The y-axes represent the number of genes in pathway analysis

To identify the pathways in which the DEGs are likely to be involved, we performed the pathway analysis with the Kyoto Encyclopedia of Genes and Genomes (KEGG) database. The formula used to determine the number of the DEGs in each pathway is the same as that used in the GO analysis. The analysis arranged 1982 DEGs to 103 KEGG pathways; top 20 pathways are shown Fig. 4. The greatest numbers of the DEGs were in metabolic pathways (268 members) and biosynthesis of secondary metabolites (205 members). The DEGs were also mapped to plant-pathogen interaction, phenylpropanoid biosynthesis and flavonoid biosynthesis.

### Comparative analysis of foxtail millet, Rice and Arabidopsis in potassium deprivation

**Fig. 5:**
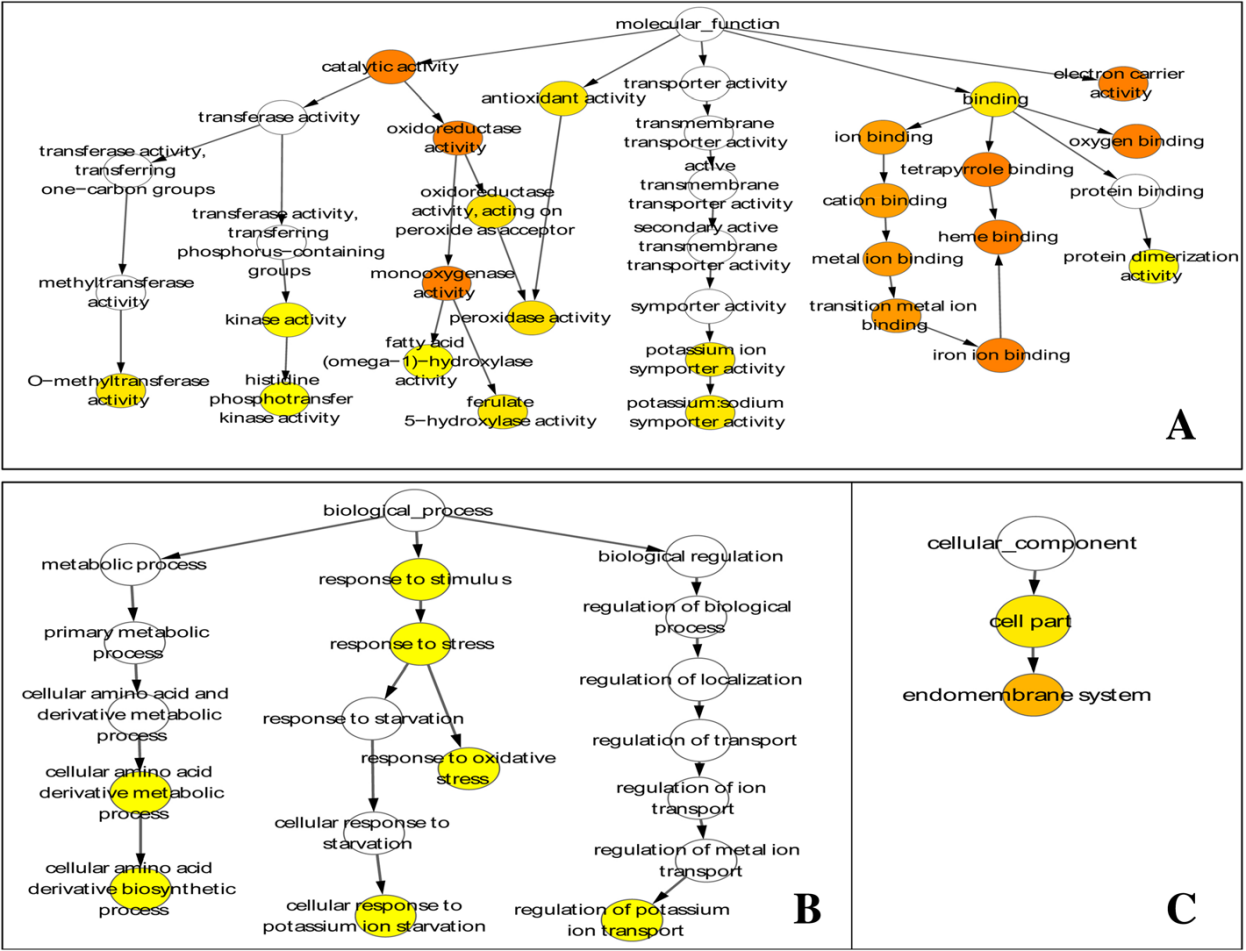
GO enrichment analysis of the potassium deprivation responding genes shared among Arabidopsis, rice, and millet. A Cytoscape view of enriched GO in biological process (**a**), molecular function (**b**), and cellular component (**c**). The colors of the node represent GO terms enriched in the targets. White nodes represent nonenriched GO terms to show the hierarchical relationship between the enriched ontology branches. Enrichment significance levels are *P*-value < 0.05 and FDR < 0.05

Transcriptome data from this study was compared with the published transcriptome data of Arabidopsis and rice on the response to potassium deficiency [11, 29]. Generally, the genes showing transcriptional changes in foxtail millet, rice and Arabidopsis in response to low-K^+^ stress displayed similar GO distribution patterns. The results indicate that genes in the categories of metabolic process may play crucial roles in responses to low-K^+^ stress among foxtail millet, rice and Arabidopsis. A total of 248 DEGs are common for potassium deprivation among foxtail millet, rice and Arabidopsis (Additional file 1: Table S6). Further GO enrichment analysis showed that some basic low potassium corresponding genetic pathways are consistent among plants under the condition of low potassium, such as binding pathway (Fig. 5), and that there may be a functional overlapping of these genes in responses to potassium deprivation in foxtail millet, rice and Arabidopsis. However, we also found that a total of 1734 DEGs was only expressed in foxtail millet under the condition of low potassium (Additional file 1: Table S7).

### RT-qPCR validation and effect of *SiMYB3* overexpression on the tolerance to K^+^ deficiency

**Fig. 6:**
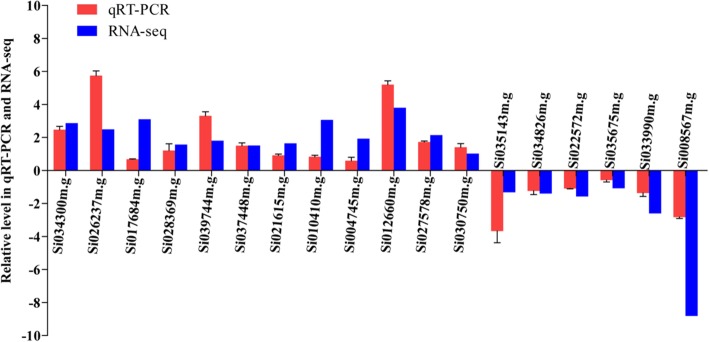
Comparison of the expressing profiles for selected DEGs between RT-qPCR analysis and RNA-Seq results. To confirm the reliability of Illumina sequencing technology, 18 candidate genes were randomly selected and their expressions were confirmed by RT-qPCR using RT-primers. Differential expression was observed for all the candidate genes, indicating that they are involved in the regulatory networks that are active during low-K stress condition. The results of RT-qPCR showed that the RT-qPCR assessments of these 18 genes were consistent with those of sequencing analysis

**Fig. 7:**
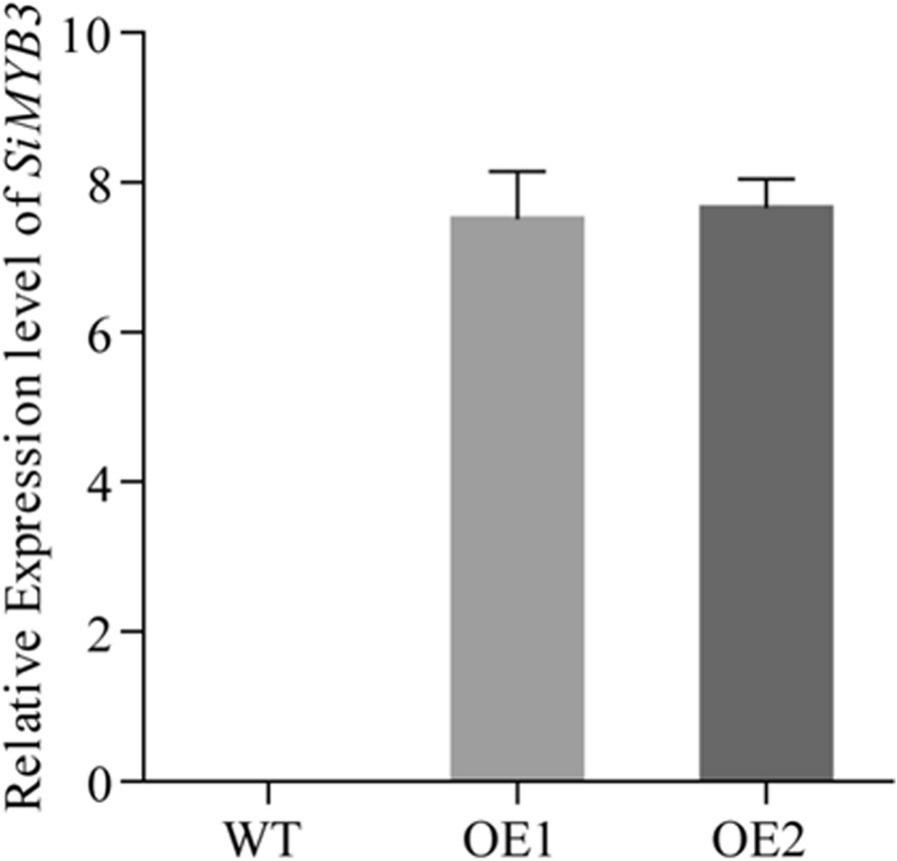
Relative expression of *SiMYB3* in the wildtype (WT), transgenic lines (OE1 and OE2) of Arabidopsis plants by RT-qPCR

To validate the DEGs, RT-qPCR analyses were performed using gene-specific primers for randomly selected 12 up-regulated and 6 down-regulated genes in the LK samples, which were identified by differential screening based on the Illumina sequencing technology (Additional file 1: Table S1). The results showed that all 18 DEGs exhibited the same expression profiles as measured in the transcriptome analysis (Fig. 6). The transcript of one gene *SiMYB3* (gene ID: Si012660m.g, the sequence of *SiMYB3* in Additional file 2: Text S1.) was highly up-regulated under the LK condition, which suggests that the expression of *SiMYB3* is induced by and responsive to the LK stress. *SiMYB3* was expressed only in foxtail millet and not in rice and Arabidopsis (Additional file 1: Table S7). Furthermore, in the T3 transgenic Arabidopsis plants, *SiMYB3* was overexpressed and detected by RT-qPCR in two lines (OE1 and OE2) but comparatively much less or no expression in the control plants (WT) (Fig. 7).

**Fig. 8:**
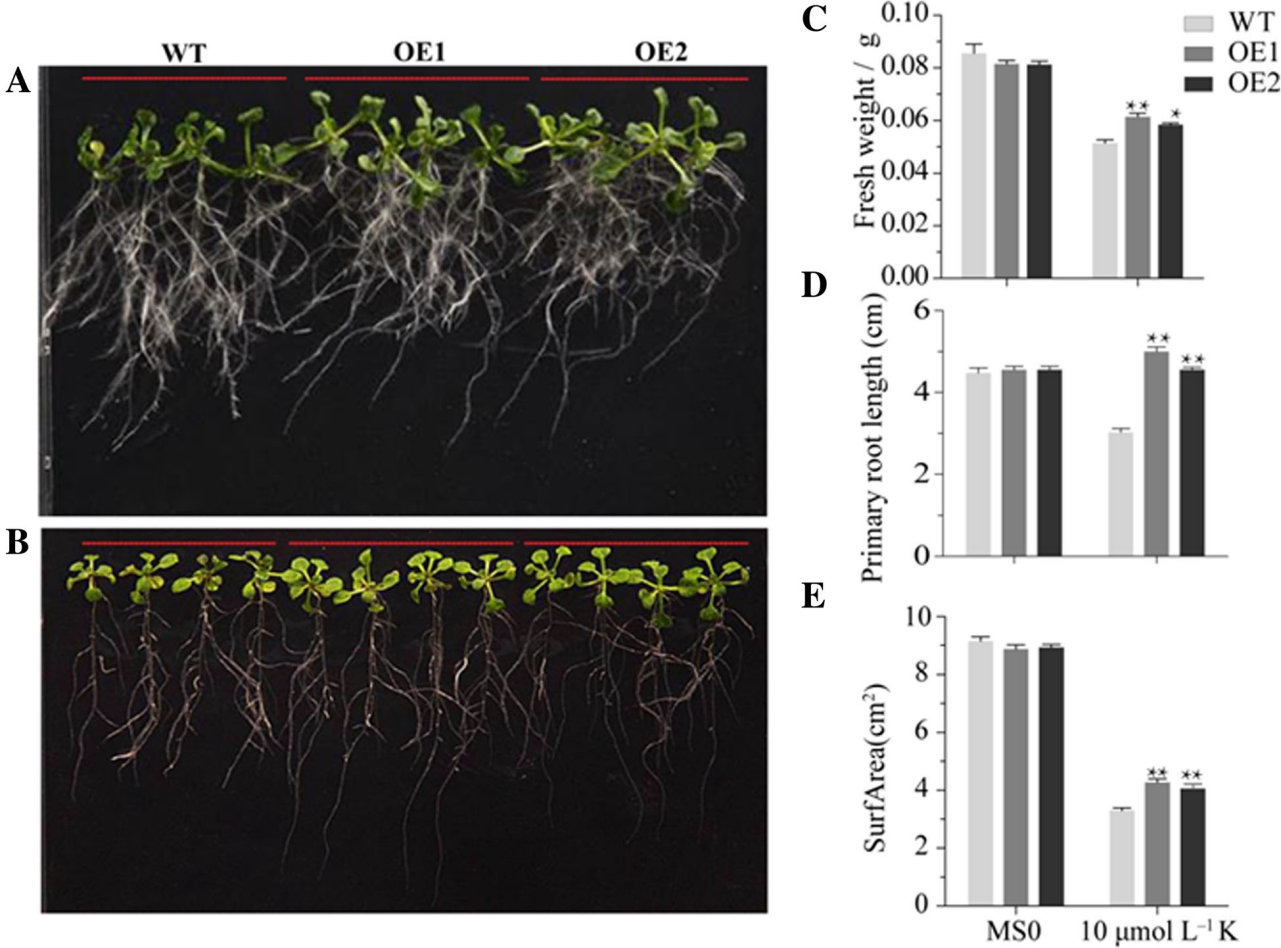
Phenotype analysis of transgenic lines during seedling stage under low potassium concentrations. **a** control; **b** Treatment under 10 μmol low potassium; **c**, **d** and **e** fresh weight, primary root length and root surface area under control and low potassium treatment, respectively. Data was statically analyzed by the means of one-way ANOVA. The * presents the significant difference at 0.05 level. The ** presents extremely significant difference at 0.01 level

To investigate whether *SiMYB3* plays a role in response to LK stress, transgenic (OE1 and OE2) and control wildtype (WT) Arabidopsis plants were subjected to CK and LK treatments. Under the CK conditions, there was no difference in development and growth between the transgenic and WT Arabidopsis plants. Under the LK conditions, the seedlings grown in potassium deficient media reduced shoot and root growth for WT Arabidopsis plants, and the transgenic Arabidopsis plants had higher fresh weight, longer primary root length and larger root surface-area than WT after K^+^ deficiency treatment (Fig. 8). These data suggest that the *SiMYB3* overexpression can promote the main root elongation and improve K^+^ deficiency tolerance in the transgenic plants. So *SiMYB3* may be involved in root development and K^+^ absorption.

## Discussion

Foxtail millet tolerates low soil fertility conditions and can achieve satisfactory yields in soils with low mineral levels [30]. However, the mechanism at transcription level underlying the tolerance against K^+^ deficiency in foxtail millet is unknown. There are very few studies reporting the gene transcriptional changes in foxtail millet for the absorption and transportation of potassium under low-K stress conditions [23]. The objective of the present study was to make a preliminary exploration at the transcript expression of foxtail millet genes under low-K^+^ stress conditions and to provide information on the mechanism behind their regulation and the transcriptomic profiling of foxtail millet seedling in response to low-K^+^ stress by use of RNA-Seq approach. Ma et al. (2012) used the GO analysis to compare and to analyze transcriptomic differences between rice and Arabidopsis under low-K^+^ stresses and found that the genes showing transcriptional changes in rice and Arabidopsis displayed similar GO distribution patterns in their responses to low-K^+^ stress [29]. In our experiments, the differentially expressed genes (DEG) were screened under CK and LK conditions. In our study, we found 1982 DEGs between the CK and LK samples, 248 DEGs are common among foxtail millet, rice and Arabidopsis, such as such as *CIPK9* (CBL-INTERACTING PROTEIN KINASE 9), *HAK5* (HIGH AFFINITY K+ TRANSPORTER 5), CYP family proteins etc. Especially *HAK5*, which is the major contributors to K+ uptake system in *Arabidopsis thaliana* [11]; And 1734 DEGs are specific to foxtail millet, including MYB, AP2, WRKY, BZIP transcription factors, etc., which may be the reason why Longgu 25 variety has good characteristics of low potassium tolerance, so the development and utilization of these genes as molecular markers can be the effective means for us to cultivate low potassium tolerance varieties. Therefore, these specific genes may be the focus of further research.

Responses against K^+^ deficiency may initiate many molecular processes such as catalytic activity, binding, transporter activity and nucleic acid binding transcription factor activity (Fig. 4). Transgenic plants produced by over-expression of functional genes have shown to be able to improve K^+^ deficiency tolerance. But, the progress in this area of research is hampered by limited availability of candidate genes with significant effect on LK phenotypes. This may be due to the lack of complete understanding of the molecular basis of K^+^ deficient tolerance and of the involvement of a complex network of genes operating in close coordination. Identifying novel genes, analyzing their expression patterns in response to K^+^ deficiency and determining their potential functions in the adaptation for K^+^ deficiency will provide the basis for effective genetic engineering strategies to enhance tolerance against K^+^ deficiency [31]. Our finding of several transcription factors in DEGs is significant. Plant-specific transcription factors play vital roles in the activation of genes associated with stress-tolerance [32]. Transcriptional control of the expression of stress-responsive genes is a crucial part of the plant response to a range of abiotic and biotic stresses. This means that transcription factors may contribute to the complexity and overlap in their responses to different stressors, and research of these factors is likely to lead to new ways of enhancing crop tolerance to environmental stress [33]. Some transcription factors are related to the development of potassium-related metabolic pathways [34]. Approximately 6 and 4% genes are transcription factors in Arabidopsis and rice, which divide into 56 and 58 transcription factor families, respectively, and the bHLH family is the largest family. In our DEGs, there are 13 families (Fig. 3, Additional file 1: Table S4), and the MYB TF family contains the largest number of DEGs (i.e., Si012660m.g, Si008086m.g, etc. Additional file 1: Table S5). The MYB transcription factors comprise of the largest transcription factor family in plants and play key roles in plant development, secondary metabolism, hormone signal transduction, disease resistance and abiotic stress tolerance [35–37]. This indicates that the foxtail millet MYB family genes may be responsive to the K-stress. Our current study identified new genes with RNA-Seq such as *SiMYB3*, a member of the MYB family, its overexpression significantly promotes the main root elongation and improves K^+^ deficiency tolerance in transgenic plants (Fig. 8). This is an extension of the function of MYB transcription factors. Because, to date, most reports suggest that MYB transcription factors can enhance the tolerance of plants to low temperature and drought. There have been few reports of its ability to increase K^+^ deficiency tolerance. For example, the overexpression of *ScT36* (an MYB-like transcription factor) in transgenic Arabidopsis resulted in enhanced cold tolerance [38], the overexpressing rice *OsMYB3R2* led to stronger cold tolerance and an increased mitotic index in transgenic rice [39], and the enhanced freezing-stress tolerance was observed in Arabidopsis overexpressing *OsMYB4* [40]. Transgenic Arabidopsis overexpressing *AtMYB15* exhibited hypersensitivity to exogenous ABA and improved drought and cold tolerance [41, 42]. Therefore, *SiMYB3* can be a useful gene in improving K^+^ deficiency tolerance for other crops. However, further research is required to explore molecular mechanisms and regulations of *SiMYB*3 under K^+^ deficiency.

In addition, 11 of 84 differentially expressed TF genes are the AP2 family proteins. Many AP2 transcription factors can activate genes in response to abiotic stress and are involved in the abscisic acid (ABA) response [43, 44]. In our study, 8 out of 10 NAC family genes (i.e., Si036535m.g, Si010053m.g, etc.) were found to be upregulated in K^+^ deficiency condition. We speculated that these genes may indirectly regulate potassium channels involved in different K-metabolic pathways in foxtail millet.

## Conclusions

Our data have substantially improved the global view of the foxtail millet transcriptome and paved the way for its further understanding of the molecular mechanisms underlying responses to K^+^ deficiency. *SiMYB3* is a valuable genetic resource of abiotic stress in foxtail millet.

## Methods

### Plant material and growth conditions

Sterilized seeds of 6 foxtail millet varieties (*Setaria italic.* cv. Longgu 25, Shiliuzi, Hongmiaodabaigu, Jigu19, Jiugu14, Changnong40) were obtained from Professor Diao Xianmin of the Institute of Crop Sciences, China Academy of Agricultural Sciences and pregerminated on moistened filter papers in a plant growth chamber under the condition of 16 h light/8 h dark (21 ± 2 °C) photoperiod with 65% humidity for 4 days. Then the seedlings were transferred into plastic pots with modified Hoagland nutrient solution (4 mM Ca(NO_3_)_2_, 6 mM KNO_3,_ 1.9 mM MgSO_4,_ 0.9 mM (NH_4_)_2_HPO_4_, 100 μM FeSO4·7H_2_O, 100 μM EDTA-2Na, 10 μM MnCl_2_, 1 μM CuSO4·5H_2_O, 1 μM ZnSO_4_·7H_2_O, 30 μM H_3_BO_3_, 0.03 μM (NH_4_)_6_Mo_7_O_24_·7H_2_O, pH 5.5). When the plants reached the 4-leaf stage, half of them were transferred to Hoagland nutrient solution with 10 μM K^+^ as the K^+^-deficient treatment (LK) supplemented with NH_4_Cl as the nitrogen source. Another half of the plants were transferred to a normal solution (6 mM K^+^) as control (CK). The nutrition solution was refreshed every 2 days. The plants were harvested in three biological replicates 10 days after the treatments for further assays.

### Measurement of biomass and K^+^ content

The foxtail millet seedlings of every variety grown in normal (CK) and potassium deficient (LK) condition were weighted (shoots and roots separately) before (fresh weight) and after (dry weight) dried at 80 °C for 48 h. The shoot-root ratio is equal to the length of shoot over the length of the root. To measure K^+^ content, the dry samples were incinerated in a muffle furnace at 575 °C for 5 h, the residue was dissolved in 0.1 N HCl, and the K^+^ content was measured by inductively coupled plasma emission spectrometer. Relative Index = X_LK_/X_CK_; where X_LK_ and X_CK_ are the total value of dry weight, ground biomass and K accumulation in shoot under LK condition and CK condition, respectively. Three biological replicates were used for the biomass and K^+^ content measurements. The t-test was used to analyze the statistical significance (* *P* < 0.05, ** *P* < 0.01).

### Preparation of cDNA library for sequencing

Fresh roots of both control and treated foxtail millet seedlings (cv.Longgu 25) were collected, immediately frozen in liquid nitrogen and stored at − 80 °C until RNA extractions. Every RNA sample was extracted from five independent seedlings. Extracted RNA was stored at − 80 °C until it was used for transcriptome sequencing and real-time fluorescent quantitative PCR (RT-qPCR) validation. Three biological replicates were used for the RNA-Seq experiments. For Illumina sequencing, the total RNA was extracted from every sample using Trizol (Invitrogen) and treated with RNase-free DNase I (TaKaRa) for 45 min according to the manufacturer’s protocol. The poly (A^+^) mRNA was purified from 20 mg of the total RNA samples using Sera-mag Magnetic Oligo(dT) Beads (Illumina). Because RNA fragmentation produces a more even sequence read distribution than cDNA fragmentation [45], the mRNA was first sheared into short fragments using an RNA fragmentation kit (Ambion) before cDNA synthesis. Using these short mRNA fragments as templates, first strand cDNAs were synthesized using random hexamer primers and the reverse transcriptase of the kit (Invitrogen). The second-strand cDNA was synthesized using DNA polymerase I, followed by the RNase H treatment to remove the RNAs. For high-throughput sequencing, the sequence libraries were constructed following the manufacturer’s instructions (Illumina). Fragments of ~ 300 bp were excised and enriched by PCR for 18 cycles. The products were loaded onto flow cell channels at a concentration of 2 pM for pair-end 90 bp × 2 sequencing. The Illumina HiSeq™ 2000 platform was used for the sequencing.

### Mapping short reads to the foxtail millet genome and the annotated gene

After removing the sequence reads containing sequencing adapters and low-quality sequence reads (reads containing more than 5% unknown bases or more than half of their bases with a quality of less than 5) using the FASTX-Toolkit software (http://hannonlab.cshl.edu/fastx_toolkit/). Biological replicates are required for almost every biological experiment, including high-throughput sequencing. If one sample is highly similar with another one, the correlation value between them is very close to 1. To assess the biological replicates, a Spearman correlation analysis of the log2-transformed FPKM values was performed. We aligned the reads to the foxtail millet genome (Phytozome v10.0.4) and annotated genes (http://brassicadb.org/brad/) using the SOAP aligner/soap2 software [46], allowing up to five mismatches. In addition, the annotated exons from the foxtail millet genome were used as reference genes to assign each read to a specific gene.

### Gene expression pattern analysis

The gene expression was calculated according to the method of RPKM (reads per kb per million reads) [47]. To identify differentially expressed genes (DEGs) between two libraries, a protocol from Audic and Claverie [48] was used. The false discovery rate (FDR) was used to determine the threshold of the *p*-value in multiple tests. The threshold of FDR ≤ 0.001 and an absolute value of log_2_Ratio ≥ 1 or log_2_Ratio ≤ − 1 were used to judge the significance of the gene expression differences [49].

The DEGs were subjected to a gene ontology (GO) analysis. For the GO analysis, the DEGs were first mapped to the GO terms in the database (http://www.geneontology.org/), the gene number was then calculated for every term, and the ultra-geometric test was used to find significantly enriched GO terms in the DEGs compared to the genome background. The *p*-value was calculated and determined using the Bonferroni correction, taking the corrected-*p*-value≤0.05 as a threshold for significance. The GO terms satisfying these conditions are defined as significantly enriched GO terms in the DEGs.

### Identification of the common pathway in response to the potassium deficiency

To investigate the common pathway in response to the potassium deficiency in plants, firstly we download the DEGs of Arabidopsis [11] and the rice cultivar Nipponbare [29] under potassium deficiency conditions, and then we aligned our DEGs against those from Arabidopsis and rice by BLASTP program with *e*-value less than 1e^− 10^. For the common DEGs, the GO enrichment analysis was carried out using the BINGO program [50] with the FDR less than 0.05.

### Real-time quantitative PCR validation of the DEGs

Real-time quantitative PCR was performed on the randomly selected eighteen DEGs in response to potassium deficiency. The extracted RNA samples were used for real-time quantitative PCR to ensure the reliability and repeatability of the results. To eliminate genomic DNA contamination, total RNA was treated with DNase I (RNase Free) (Takara, Dalian, China) and then used to synthesize cDNA by a reverse transcription reaction using random primers (Promega, Madison, WI, USA). Three biological replicates were performed using the Power SYBR Green PCR Master Mix (Applied Biosystems, Foster City, CA, USA) on the 7500 Real Time PCR System machine (Applied Biosystems) according to the manufacturer’s protocols. The PCR-cycling conditions comprised of an initial polymerase activation step at 95 °C for 5 min, followed by 40 cycles at 95 °C for 10 s, 60 °C for 20 s and 72 °C for 32 s. After each PCR run, a dissociation curve was measured to check the specificity of the product and the production of primer dimers. The relative amounts of the amplified products were calculated based on the standard curves generated by using standard cDNAs of known concentrations. The gene-specific primers (Additional file 1: Table S1) were designed using DNAMAN. The amplification of *SiActin* (*Si001873m.g*) was used as an internal control to normalize the data.

### Cloning of SiMYB3

Total RNA was isolated with TRIzol reagent (Invitrogen, Carlsbad, CA) from 14-d-old seedlings of Longgu 25 variety. RNA quality and quantity were analyzed by electrophoresis and spectrophotometry. For cDNA synthesis, 1 μg of total RNA was used for reverse transcription with FastKing RT Kit (with gDNase) (Tiangen) according to the manufacturer’s recommendations. The coding region sequence of *SiMYB3* with a total size of 948 bp was amplified with the PrimeSTAR DNA polymerase PCR kit (Clontech Takara) and primers 5’-TAGAGGATCCCCGGGATGGGGAGGTCGCCGT-3′ and 5’-AGTGGATCCCCCGGGTCATAGTGGCAAGCTG-3′. The fragment obtained was subcloned into pBI121 at SmaI site, and constructs were verified by sequencing.

### Generation of transgenic Arabidopsis plants and analysis of the LK tolerances

*Arabidopsis thaliana* ecotype Columbia-0 was used as the wild type (WT) for all experiments carried out in this study. For phenotypic analysis, if not indicated differently, plants were cultivated in a growth room with long-day growth conditions (16 h white light, 80–100 μmol•m-2•s^− 1^/8 h dark) at 21 ± 2 °C. To construct expression vectors for the transformation of Arabidopsis plants, *SiMYB3* gene was ligated into the vector pBI121 under control of the CaMV35S promoter. Flower-budding Arabidopsis Col-0 plants were transformed using the floral dip method under vacuum conditions as described previously [51]. T3 transgenic plants were identified by RT-qPCR and repeated with at least three independent plants. Primers were 5′-GGCAAACAGGGAGAAGATGA-3’and 5′- GGCAAACAGGGAGAAGATGA-3′ for actin; 5′-AACTGAACGCCGCTTCATC-3’and 5′- GCTGCTCTCTGCTAGCATGTC-3′ for *SiMYB3*. The reaction was performed under the following conditions: 3 min denaturation at 94 °C; then 40 cycles of 10 s at 94 °C, 20 s at 60 °C and 32 s at 72 °C. Then positive transgenic plants were used for further analysis. Transgenic and wildtype (WT) Arabidopsis plants were subjected to LK treatments and control (MS0). Fifty plants of each line were used for the stress treatment. And then plants were subjected to the measurements of fresh weight, primary root length and root surface-area.

## Additional files

Additional file 1: **Table S1.** List of the primers used in qRT-PCR analyses. **Table S2.** List of all genes showing transcriptional changes during K + −deficiency. **Table S3.** The differentially expressed genes (1982 genes) for the K^+^ deficient stress in foxtail millet. **Table S4.** Gene ontology (GO) classification of the differentially expressed genes. **Table S5.** Detail of differentially expressed transcription factors. **Table S6.** List of 248 differentially expressed genes common for potassium deprivation among millet, rice and Arabidopsis. **Table S7.** List of 1734 specifically expressed genes in foxtail millet for potassium deprivation. (XLSX 3089 kb)

Additional file 2: **Text S1.** The sequence of ***SiMYB3***. (DOCX 11 kb)

## Abbreviations

AP2: APETALA2
BGI: the Beijing Genomics Institute
bHLH: Basic Helix-Loop-Helix
bZIP: Basic region leucine zipper
CK: Control
DEGs: Differentially expressed genes
FDR: The false discovery rate
GO: Gene Ontology
JGI: China-US Department of Energy-Joint Genome Initiative
K: Potassium
KEGG: the Kyoto Encyclopedia of Genes and Genomes
LK: Potassium deficient
MYB: v-myb avian myeloblastosis viral oncogene homolog
NAC: NAM/ATAF1/2/CUC1/2
RPKM: Reads per kb per million reads
TF: Transcription factor
WT: Wildtype
