# Supplementary material for: Genome-wide analysis and identification of the low potassium stress responsive gene SiMYB3 in foxtail millet (Setariaitalica L.)
Source: BMC Genomics. 2019 Feb 15;20:136. doi: 10.1186/s12864-019-5519-2 (PMC6377754; doi:10.1186/s12864-019-5519-2)
Supplement: Supplementary file 2 — Text S1. The sequence of SiMYB3. (DOCX 11 kb) [file 12864_2019_5519_MOESM2_ESM.docx]

>Si012660m.g   CDS

ATGGGGAGGTCGCCGTGCTGCTGCCACGACGCCGGCGTGAAGAAAGGCCCGTGGACGGAGGAGGAGGATCGGGCGCTGGTGGAGCACATCCAGAGGCACGGCGGGCACGTCGGCAGCTGGCGCAACCTGCCCAAGGCCGCCGGGCTGAACCGCTGCGGCAAGAGCTGCCGCCTCCGCTGGACCAACTACCTCCGCCCCGACATCAAGCGCGGCAACTTCACCGACGACGAGGAGCGCCTCATCATCGCCCTCCACGCCGAGCTCGGCAACAAGTGGTCGACGATCGCGACGCACCTGGACGGCCGGACGGACAACGAGATCAAGAACTACTGGAACACGCACATCCGGAAGAAGCTGCTGCGCATGGGCGTCGACCCCGTCACGCACCAGCGGCTGCCCCCTGACGACATCCTCGCCTCCGCCGCCGGCGCCCCCGGCCTCTCCGAGGCGCTCCTCTCGGCGGCGGCTAGCCTCGGAGGCCTCAACAACGTCCTGATGCAGGTGCAGGCGCTGCAGCTCCTGCTGCAGACCATCAACGGAGGCGCCGCTGCAGCTGGTCTCATGGCCAATAATAACTTCGGCTCGGCTGCAGACAACAACGCCATGTTTAACGCAAGGAGCATGGTTCCAAACTTCCAGGACCAGATGAACCTCTTGGCTCACGCGAACTACCGGCCGGTTGACGATTATCTCAACAATATAGCAAGTTTTTCAGAGCACGACGCGGTGCAGCAACTGAACGCCGCTTCATCAGCTCCGGCGCCGACTGCGGCGGCATTGGCGGTGCCTGCGTCTTTTCCGCAGGAAGTGGCAGCTGCAGCTGACCGGCCGGTGCAGGGTTTCGCCGATCTTCTATCGGAGGCCAATGAGATGCCGAACATGTGCTCTCTGGAGGATGATCGTTTCTGGAAAGACATGCTAGCAGAGAGCAGCAGCTTGCCACTATGA
